# Recombinant avian metapneumovirus subtype C expressing HA protein of H9N2 avian influenza virus are stable and induce protection

**DOI:** 10.3389/fmicb.2024.1513474

**Published:** 2024-12-18

**Authors:** Yu Guo, Jing Cheng, Shuai Zhang, Yuanyuan Zhang, Yuzhu Zuo, Tao Liu, Yun Wang, Chun Yang, Chunjie Cheng, Jinghui Fan, Haijun Jiang

**Affiliations:** ^1^The College of Veterinary Medicine, Agricultural University of Hebei, Baoding, China; ^2^Institute of Animal Husbandry and Veterinary Medicine, Beijing Academy of Agriculture and Forestry Sciences, Beijing, China; ^3^Baoding Vocational and Technical College, Baoding, China

**Keywords:** avian metapneumonovirus/C, H9N2 avian influenza virus, enhance green fluorescent protein, HA protein, immune protection

## Abstract

To prevent H9N2 avian influenza virus (AIV) and Avian metapneumonovirus/C (aMPV/C) infections, we constructed recombinant aMPV/C viruses expressing the HA protein of H9N2 AIV. In addition, EGFP was inserted into the intermediate non-coding region of P-M protein in the aMPV/C genome using a reverse genetic system. The conditions for rescuing the recombinant virus were enhanced followed by insertion of the H9N2 AIV HA gene into the same location in the aMPV/C. The constructed recombinant virus raMPV/C-HA expressed the H9N2 AIV HA protein and showed good stability. Immunization of chicks with raMPV/C-HA increased the generation of neutralizing antibodies against aMPV and H9N2 AIV for 21 days. In the late challenge experiment, raMPV/C-HA effectively inhibited the replication of the virus *in vivo*, decreased the incidence of infection and conferred protection effects.

## 1 Introduction

*Avian metapneumovirus* (aMPV) is member of the genus Metapneumovirus belonging to the *Pneumovirinae* subfamily of *Paramyxoviridae* (Cui et al., 2024). It is a non-segmented, single-stranded, negative-strand RNA virus with a genome length of approximately 13.5 kb. The aMPV genome encodes eight proteins, and its structure from 3′ to 5′ is 3′ -leader-N-P-M-F-M2-SH-G-L-trailer-5′ (Cui et al., 2024).

The aMPV virus has been shown to cause acute respiratory infections and affect egg production in commercial turkeys. In addition, it has been shown to induce the swollen head syndrome in broilers and breeding chickens (Franzo et al., 2024). The virus is classified into four subtypes: A, B, C, and D (Goraichuk et al., 2024). Among these, subtype C exhibits significant differences from the other three subtypes but shares a higher degree of homology with human metapneumovirus (hMPV). The ability of aMPV/C to cause mice infections suggest that it may also infect humans (Cui et al., 2024; Wei et al., 2014).

Different subtypes of aMPV have been detected in farmed and wild birds worldwide (Goraichuk et al., 2024; Sun et al., 2014). Despite the development of vaccines against aMPV, several field virus strains have evolved to avoid vaccine-induced immunity (Catelli et al., 2010; Cecchinato et al., 2010). Therefore, it is imperative to formulate new vaccines to control aMPV-associated infections.

The H9N2 avian influenza virus (AIV), a member of the influenza virus A genus of the *Orthomyxoviridae* family, exhibits low pathogenic AIV. Although it only causes mild respiratory diseases in chickens, it can potentially decrease immunity in chickens, and hence, secondary infections (Wen et al., 2024; Dong et al., 2022).

H9N2 is currently the most prevalent AIV in China, and has potential for cross-species transmission, and cases of H9N2 AIV infection of pigs, dogs, and even humans have been reported (Sun and Liu, 2015; Cong et al., 2007; Sun et al., 2013; Song and Qin, 2020). Currently, the H9N2 AIV infection is prevented mainly using inactivated vaccines, but the continuous variability of the antigenicity of H9N2 AIV is major hurdle to the effective prevention of infections (Dong et al., 2022). The hemagglutinin (HA) gene encodes a glycoprotein expressed on the surface of AVI virus membrane, and also serve as an immune antigen in the body that elicits the production of neutralizing antibodies (Yang et al., 2023). The HA protein is cleaved into HA1 and HA2, with HA1 containing receptor binding sites and most of the antigenic determinants that recognize neutralizing antibodies. HA2 facilitates the fusion of viral envelope with the host cell and induce cross-protection in the body (Wang et al., 2022).

As a member of the *paramyxoviridae* family, the aMPV genome can be inserted as a foreign gene to construct recombinant viruses, like Sendai virus (Zhan et al., 2015), Newcastle disease virus (He et al., 2023), and respiratory syncytial virus (Wang et al., 2021). Several techniques have been developed to facilitate the insertion and deletion of aMPV genomes using reverse genetic techniques (Naylor et al., 2004; Naylor et al., 2010; Govindarajan et al., 2006). However, few studies have investigated designed recombinant viruses by inserting aMPV into foreign virus genes.

In this study, we constructed a mini genome expressing EGFP to test the functionality of the aMPV reverse genetic system. Subsequently, the EGFP and HA genes were inserted between the P and M genes of aMPV leading to the construction and rescue of the recombinant aMPV viruses expressing EGFP and HA. Finally, to assess the immunological efficacy of raMPV/C-HA in chickens, immunized birds were challenged with both aMPV and H9N2 AIV.

## 2 Materials and methods

### 2.1 Cell line, virus, plasmids and nucleic acid

Baby hamster kidney (BHK-21) cell line and Vero African green monkey kidney (vero) cells were freely donated by Prof. Zhenhua Zhang (Beijing Academy of Agriculture and Forestry Sciences, Beijing, China). The cells were cultured in the DMEM (Gibco, Grand Island, United States) containing 10% fetal calf serum and antibiotics (100 U/mL penicillin, 100 mg/mL streptomycin, 0.25 mg/mL amphotericin B, Thermo Scientific).

The modified vaccinia Ankara/T7 recombinant virus (MVA/T7) was donated by B. Moss, National Institutes of Health. The reverse genetics system of aMPV/C (accession number: AY579780.1) and helper plasmids (pcDNA3.1-N, P and L) expressing the N, P, and L proteins of aMPV/C were kindly provided by Prof. Qingzhong Yu (SEPRL, USDA, USA). The pcDNA3.1-EGFP plasmid was kept in our laboratory. The challenge experiments were conducted using aMPV/C (AY590688) and H9N2 AIV (MK552998) strains, kept in our laboratory. The H9N2 AIV HA gene was synthesized by the company (Beijing Tsingke Biotech Company, Beijing, China).

### 2.2 Construction of minigenome rescue system and recombinant virus

The aMPV/C minigenome rescue system, containing EGFP, was constructed using the p-bluescript. The p-bluescript vector contains T7 promoter, terminator and hepatitis delta virus ribozyme (HDVRz) downstream.

**Figure 1:**
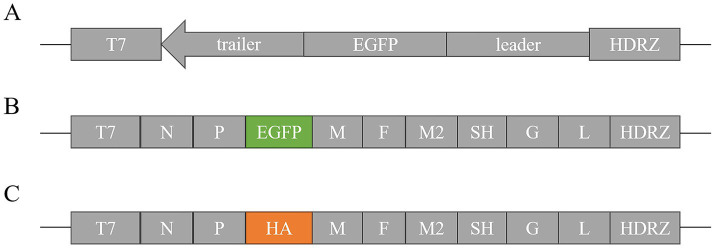
Structure of pBR-miniaMPV/C, raMPV/C-EGFP and raMPV/C-HA. **(A)** Construction of pBR-miniaMPV/C: Leader-EGFP-Trailer was cloned into the p-bluescript vector and inserted between the T7 promoter and HDVRz. **(B)** Construction of raMPV/C-EGFP: The EGFP gene was inserted between the P and M proteins of the ampv genome. **(C)** Construction of raMPV/C-HA: The AIV H9N2-HA gene was inserted between the P and M proteins of the ampv genome.

The aMPV-leader region (nt 1–53), aMPV-trailer region (nt 13,970–14,150) and EGFP gene fragments were amplified using aMPV and pcDNA3.1-EGFP plasmid. The aMPV-leader, EGFP and aMPV-trailer were seamlessly cloned into the p-bluescript vector in a reverse complementary direction. The pBR-miniaMPV/C minigene rescue system was developed as presented in Figure 1. The success of insertions into the minigenomes were verified through DNA sequencing.

The EGFP, and HA genes with aMPV transcriptional signals were amplified by PCR and inserted into the non-coding region of the P-M gene of aMPV through seamless cloning. Construction procedure of raMPV/C-EGFP and raMPV/C-HA were presented in Figure 1. The EGFP and HA insertions into the aMPV genome were confirmed via the sequencing the PCR products of the viral genomes, which confirmed the nucleotide sequence fidelity of the rescued viruses.

### 2.3 Transfection

The recombinant virus plasmid (pBR-miniaMPV/C/raMPV/C-EGFP and raMPV/C-HA) and three helper plasmids (N, P, and L) were extracted using the QIAGEN Plasmid Midi kits (Qiagen, Germany) following the manufacture’s protocol. In summary, when the BHK-21 cells cultured in a six-well plate grew to approximately 80% confluence, 5 μg of recombinant virus plasmid, 2 μg of pcDNA3.1-N, 2 μg of pcDNA3.1-P and 1 μg of pcDNA3.1-L were co-transfected using LipofectamineTM 3000 (Invitrogen, Carlsbad, CA, United States) in line with the manufacturer’s instructions. Cells in the negative control were transfected as described above, with the except the pcDNA3.1-L plasmid. BHK-21 cells were infected with MVA/T7 at a multiplicity of infection (MOI) of 1 to generate T7 polymerase, 1 h prior to transfection. After transfection, the cells were incubated for an additional 72 h at 37°C in a humidified incubator with 5% CO_2_. Cells transfected without helper plasmids served as negative controls.

During transfection, the EGFP expression in pBR-miniaMPV/C and raMPV/C-EGFP was examined using an inverted fluorescence microscopy (Leica, Germany).

Seventy-two hours post-transfection, when approximately 80% of the cells exhibited cytopathic effects, the cells were subjected to three cycles of freezing and thawing and subsequently stored at −80°C. The harvested recombinant viruses (raMPV/C-EGFP and raMPV/C-HA) were inoculated into Vero cells. Following a 1-h infection period, the viral inoculum was removed and replaced with DMEM containing 2% FBS. Once 80% of the cells displayed cytopathic effects, they were again frozen and thawed three times. This process was employed for viral passage and propagation.

### 2.4 The replication capacity of recombinant virus

The recombinant virus (raMPV/C-EGFP and raMPV/C-HA) was diluted 10 times repeatedly, and 100 μL was used to infect Vero cells for the determination of the viral titer (Kapczynski and Sellers, 2003). Titers were calculated using the Reed and Muench method and are presented as 50% tissue culture infective dose (TCID_50_) (Reed and Muench, 1938). Vero cells were infected with aMPV/C, raMPV/C-EGFP and raMPV/C-HA at 0.1 an MOI. Monolayer cells were collected every 4 h after infection. The mean titers of different viruses at each time point were calculated and expressed as the log_10_ TCID_50_/mL.

### 2.5 Determination of genetic stability of the recombinant virus

#### 2.5.1 Virus sequencing

The raMPV/C-EGFP and raMPV/C-HA were passaged to 20 generations in vero cells, and 5, 10, 15, and 20 generations of the virus were collected to extract RNA. The RNA was reverse-transcribed into cDNA, and the foreign gene (EGFP/HA) and the N gene of aMPV were the quantified through the PCR test. All PCR products were sequenced by a company (Beijing Tsingke Biotech Company, Beijing, China) to ensure that the sequence was mutation-free.

#### 2.5.2 Western blot and indirect immunofluorescence assay

Indirect immunofluorescence assay (IFA) and Western blot confirmed that recombinant viruses (raMPV/C-EGFP and raMPV/C-HA) of different generations (Sun et al., 2014; Sun and Liu, 2015; Wang et al., 2022; Naylor et al., 2010) expressed HA and EGFP.

### 2.6 Animal experimental design

One-week-old SPF grade chickens were randomly divided into 7 groups with 10 chickens per group. The chickens in groups 1 and 2 were vaccinated intranasally with 2 × 10^5^ TCID_50_ of raMPV/C-HA, and those in groups 3 and 4 aMPV/C were vaccinated intranasally with 2 × 10^5^ TCID_50_ of aMPV/C. After 4 weeks of immunization, chicken in groups 1, 3, and 5 were challenged with aMPV/C (2 × 10^6^ TCID_50_). Those in groups 2, 4, and 6 received intramuscular injection of H9N2 AIV (2 × 10^6^ EID_50_). Each chicken in group 7 was vaccinated intranasally with 100 μL DMEM. Those in groups 1–4 were served as the immune experimental group, whereas 5 and 6 were the unimmunized groups but challenged positive controls, and group 7 served as the negative controls. After inoculation, the clinical symptoms of chickens were recorded. Serum samples were collected from chickens in each group on days 21 and 28 post-immunization to assess the antibody response. Tracheal swabs were obtained from each chicken on days 3, 5, and 7 post-challenge to evaluate viral replication or viral RNA shedding in the respiratory tract. Each swab was stored in 1 mL of DMEM medium containing antibiotics at −70°C.

### 2.7 Determination of neutralizing antibody

The anti-aMPV neutralizing antibody titers were calculated using the plaque reduction neutralizing assay, while the H9N2 AIV neutralizing antibody titers were determined using Hemagglutination inhibition (HI) and neutralization test (NT). The detailed experimental assays were described previously (Wei et al., 2014; Sun et al., 2014; Nagy et al., 2016; Tang et al., 2013).

### 2.8 Quantification of viral genomic RNA by quantitative real-time PCR

Quantitative real-time PCR (qRT-PCR) was used to quantitatively detect aMPV and H9N2 AIV virus concentrations in swabs (Cecchinato et al., 2013). The primers used for qRT-PCR are provided in previous studies (Falchieri et al., 2013; Shao et al., 2022).

### 2.9 Statistical analysis

All the experiments were repeated at least three times, and the results were expressed as the mean ± SE. Statistical analyses were performed using the SPSS software package V11.5 (SPSS Inc., Chicago, IL). All data were compared among the groups using one-way analysis of variance (ANOVA). A *p* < 0.01 was considered statistically significant.

## 3 Results

### 3.1 Generation of aMPV-minigenome

**Figure 2:**
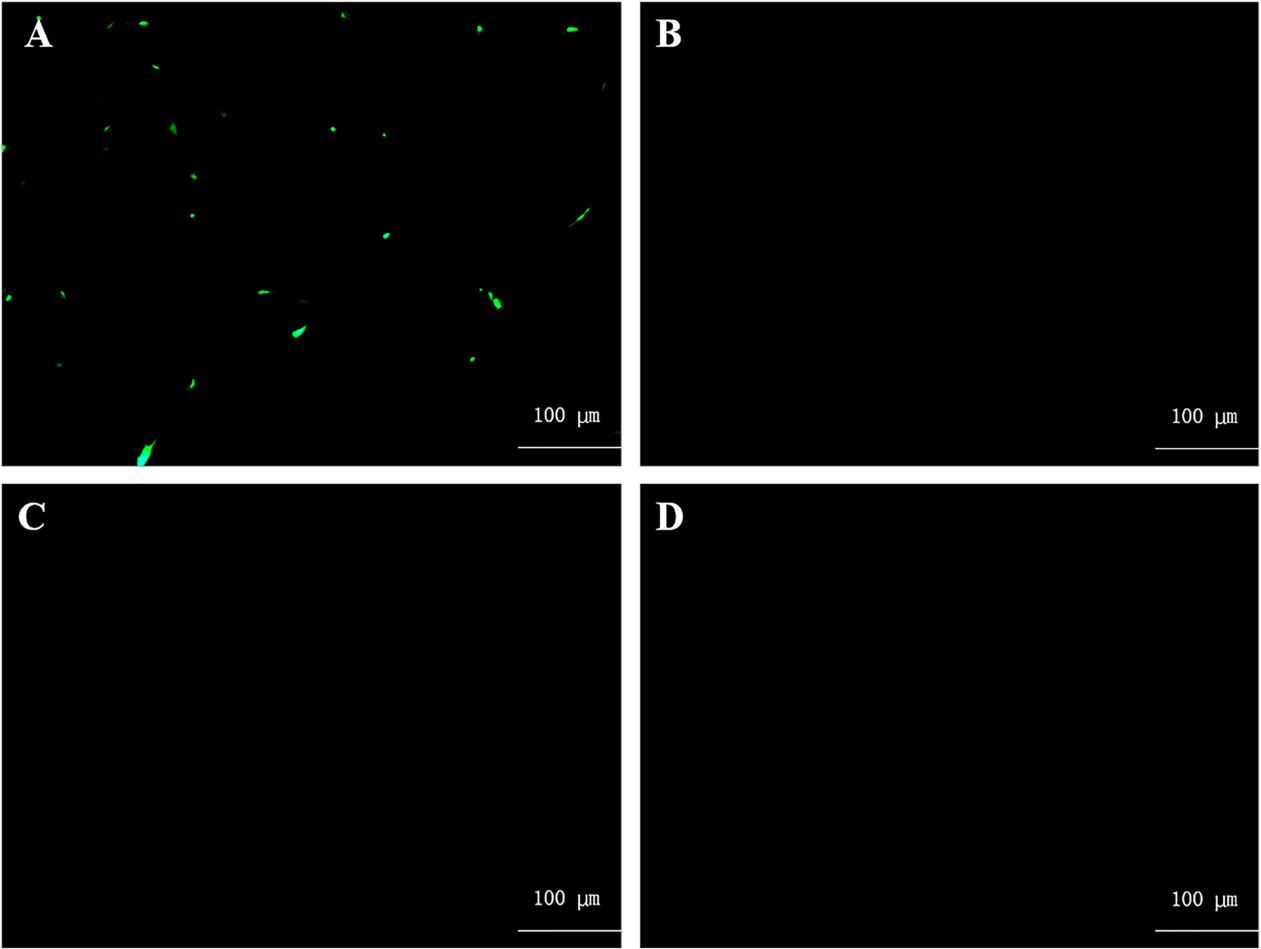
Rescue of pBR-miniaMPV/C minigenome. **(A)** BHK-21 cells were co-transfected with pBR-miniaMPV/C, pcDNA3.1-N (PN), pcDNA3.1-P (PP), and pcDNA3.1-L (PL) plasmids. The MVA/T7 was infected 1 h before transfection. **(B)** BHK-21 cells were co-transfected with pBR-miniaMPV/C, PN, PP, and PL plasmids without MVA/T7 infected before transfection. **(C)** BHK-21 cells were co-transfected with pBR-miniaMPV/C, PN and PP, but without PL. The MVA/T7 was infected 1 h before transfection. **(D)** BHK-21 cells were transfected with PN, PP, and PL plasmids, without pBR-miniaMPV/C. The MVA/T7 was infected 1 h before transfection. EGFP expression was examined 48 h post-infection. Scale bar 100 μm.

Following a 72-h transfection of pBR-miniaMPV/C and three helper plasmids into BHK-21 cells, pBR-miniaMPV/C expressed green fluorescent protein which was absent in the negative control group (Figure 2).

### 3.2 Analysis of the replication capacity of recombinant virus

**Figure 3:**
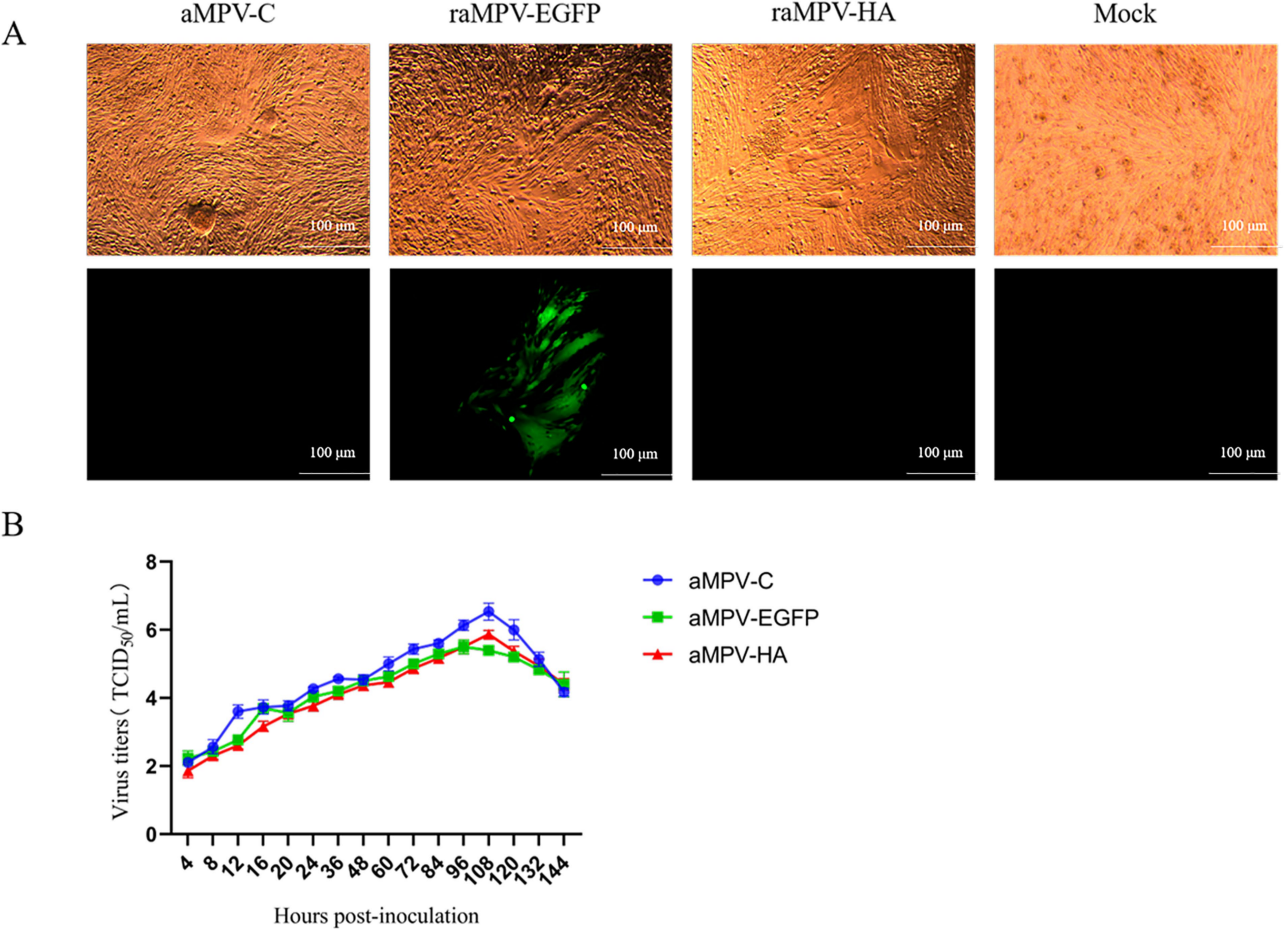
Characterization of the rescued parental virus (aMPV/C) and recombinant virus (raMPV/C-EGFP and raMPV/C-HA). **(A)** The cytopathic effects induced by aMPV/C, raMPV/C-EGFP and raMPV/C-HA. Scale bar 100 μm. **(B)** Multistep growth curve of aMPV/C, raMPV/C-EGFP and raMPV/C-HA in cell cultures. Data presented are the means ± standard deviations (S.D.) from three independent experiments.

The raMPV/C-EGFP and raMPV/C-HA and three helper plasmids were co-transfected into BHK-21 cells and amplified in the Vero cells. The expression of EGFP and cytopathic effects (CPE) were detected using a fluorescence inverted microscope (Figure 3A). Both raMPV/C-HA and raMPV/C-EGFP infections exhibited similar cytopathic effects (CPE) to those observed with the parental strains. Analysis of the viral growth curve revealed that while the recombinant virus exhibited slightly lower replication rates compared to the parental strain, this difference persisted throughout most of the infection period (Figure 3B).

### 3.3 The genetic stability of recombinant virus

RNA was extracted and from recombinant viruses of different generations and sequenced. The exogenous genes of EGFP and HA were stably inserted between the P and M genes in raMPV/C-HA and raMPV/C-EGFP.

**Figure 4:**
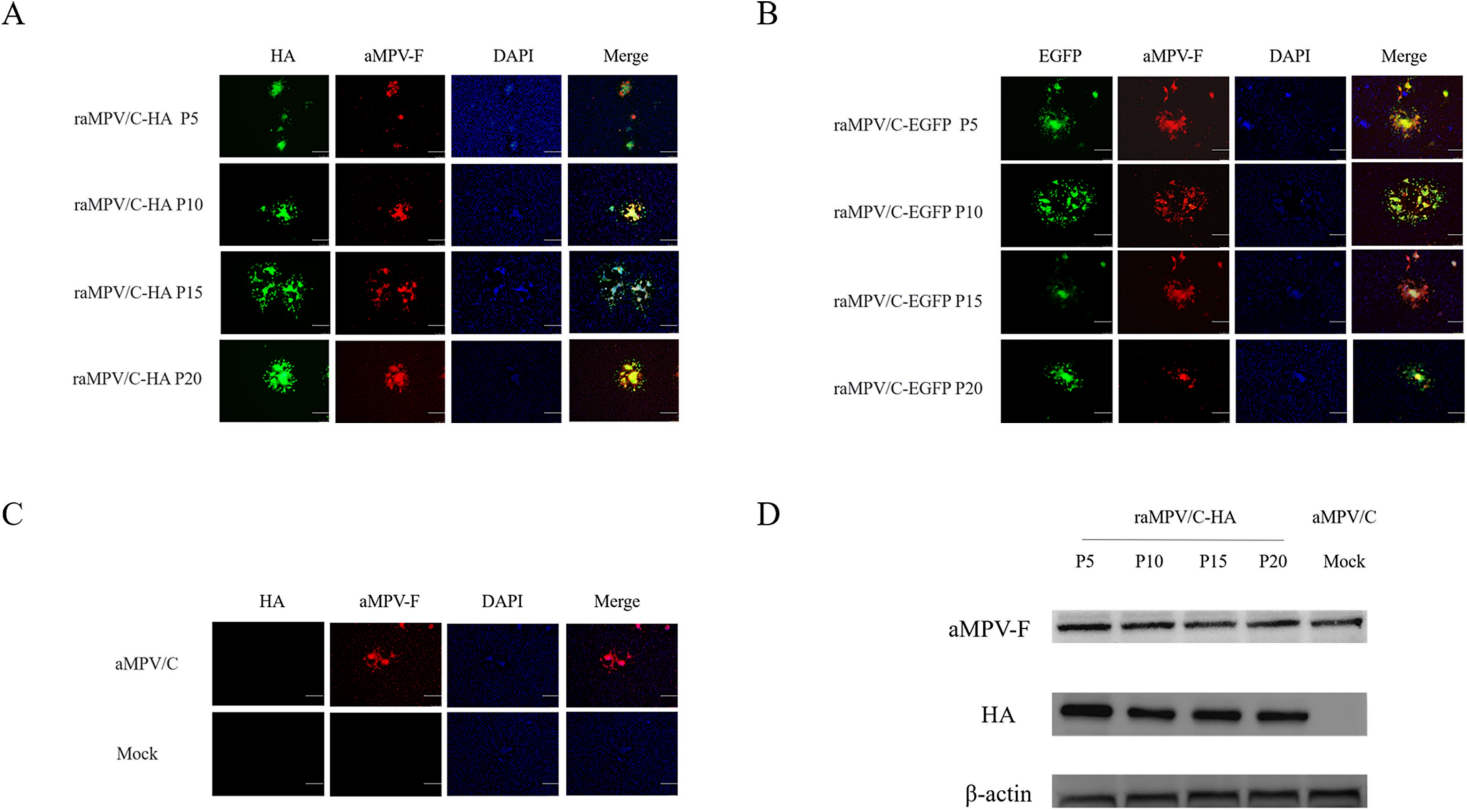
Characterization of the raMPV/C-EGFP and raMPV/C-HA. IFA of raMPV/C-HA **(A)**, raMPV/C-EGFP **(B)**, parental aMPV/C and mock (PBS) **(C)** infected Vero cells at 48 h using anti-aMPV-F and HA monoclonal antibody. The bar = 100 μm. **(D)** Western blotting was used to detect the expression of HA and aMPV-F in raMPV/C-HA. Parental aMPV/C was negative control. Chicken *β*-actin was detected as the internal control.

Indirect immunofluorescence assay (IFA) indicated that all generations of raMPV/C-EGFP reacted with aMPV-F monoclonal antibody (Figure 4B). Notably, the raMPV/C-HA reacted with the aMPV-F monoclonal antibody and HA monoclonal antibody (Figure 4A). The raMPV/C-EGFP and raMPV/C-HA strains expressed EGFP and HA proteins, respectively. The parental aMPV/C virus infection group only reacted with AMPV-F monoclonal antibody, and the PBS infection group did not react with any antibody (Figure 4C). Results of Western Blot (WB) confirmed that HA (63 kDa) was strongly expressed (Figure 4D).

### 3.4 Antibody responses against aMPV and HA induced by raMPV/C-HA in chickens

**Figure 5:**
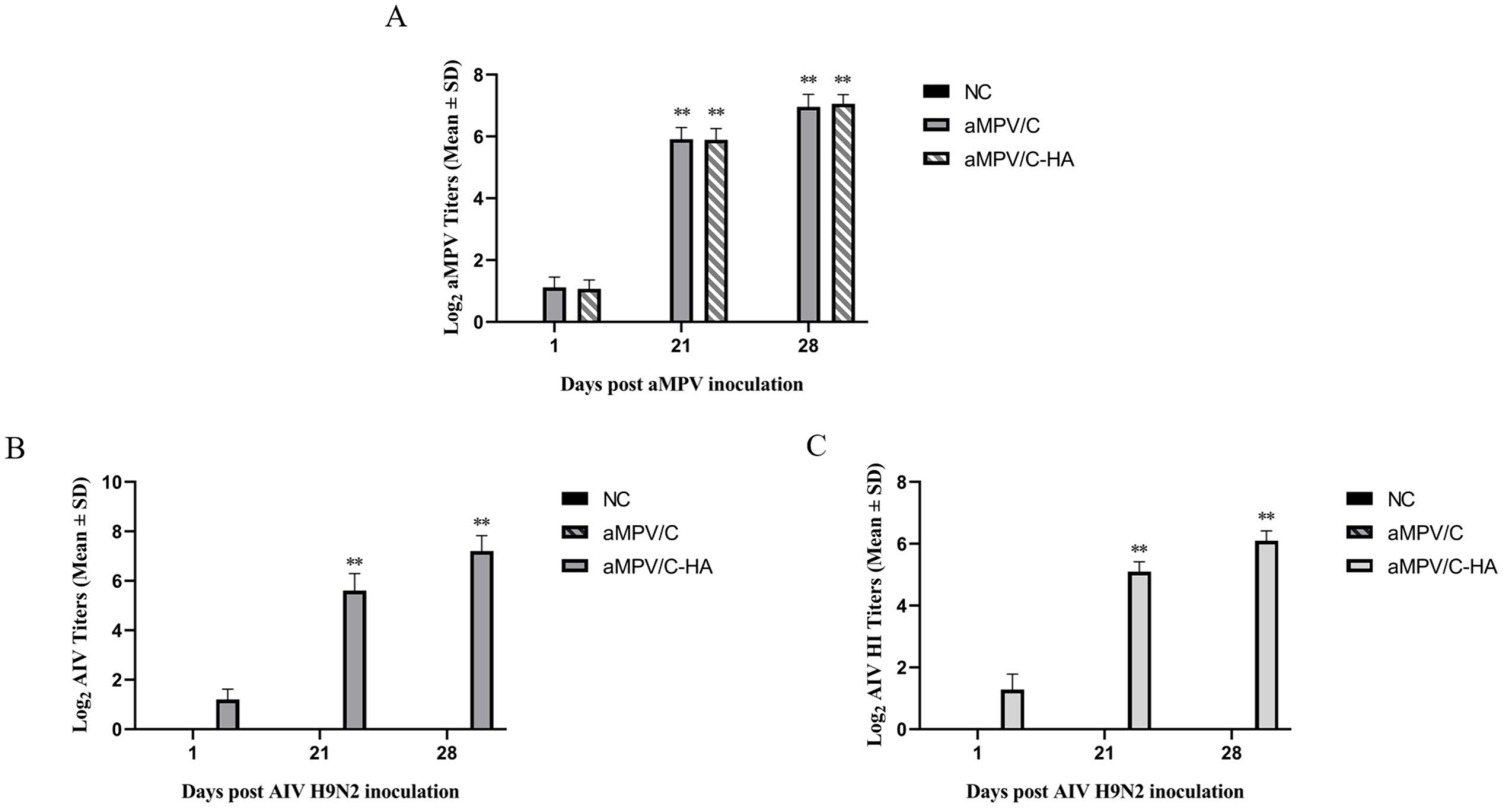
aMPV/C and raMPV/C-HA induced the generation of high titers of neutralizing antibody in chickens. **(A)** The concentration of neutralizing antibody titer against aMPV in serum samples collected from chicken at 1, 21, and 28 dpi. Hemagglutination inhibition (HI) **(B)** and neutralization test (NT) **(C)** were conducted to detect the anti-AIV titers in chicken serum at 1, 21, and 28 dpi. NC: The negative control group received PBS through nose drops. Data are shown as the mean ± standard deviation (S.D.) from 10 birds in each group. The differences between groups were analyzed using ANOVA (**p < 0.01).

At 21 d after inoculation, the aMPV/C and raMPV/C-HA groups produced significant amounts of the neutralizing antibodies against the aMPV compared with the control group (*p* < 0.01). In addition, the content of the aMPV neutralizing antibody in serum of chickens at 28 d after inoculation was also higher compared with that at 21 d (Figure 5A).

In this study, two methods of HI and NT were used to detect anti-AIV antibody titers in serum. As expected, no neutralizing antibodies against HA were generated by aMPV/C and the control group at 21 d and 28 d of inoculation. However, serum levels of anti-Ha-neutralizing antibodies in raMPV/C-HA vaccinated chickens increased significantly at 21 and 28 d (*p* < 0.01). The results of HI and NT antibody titer against AIV were consistent (Figures 5B,C).

### 3.5 Protection efficacy against aMPV and H9N2 AIV challenge in chickens

The chickens challenged with aMPV and H9N2 AIV on the 28th day of immunization. In the groups challenged with aMPV, all the non-immunized chickens became ill at 3 days post-inoculation (dpi), and the infection gradually decreased to 50%. Generally, the clinical symptoms of aMPV infection are detected at the onset, including cough, swollen infraorbital sinuses and turbid nasal exudates. Compared with the positive control group, chickens in the immunized group had a lower incidence (10–20%), mild clinical symptoms, and no significant local swelling and cough (Table 1).

**Table 1:** Observation of clinical signs of birds at different days post-challenge (DPC).

Challenge experiment	Groups	Number of birds showing clinical signs/number in group		
		3 DPC	5 DPC	7 DPC
aMPV/C	aMPV/C	1/10	1/10	0/10
	aMPV/C-HA	2/10	1/10	0/10
	C+	10/10	9/10	5/10
	C−	0	0	0
AIV H9N2	aMPV/C	9/10	7/10	5/10
	aMPV/C-HA	1/10	0/10	0/10
	C+	10/10	8/10	6/10
	C−	0	0	0

For the H9N2 AIV challenge, raMPV/C-HA exhibited almost complete protection to the flock, with one chicken exhibiting mild symptoms at 3 dpi which turned negative at 5 dpi. Almost all aMPV/C vaccinated chickens (90%) and unvaccinated chickens (100%) developed clinical symptoms on 3 dpi, and the incidence remained above 50% until 7 dpi. Moreover, both groups exhibited the typical clinical symptoms of H9N2 AIV, including coughing, sneezing, rales, and excessive tearing (Table 1).

**Figure 6:**
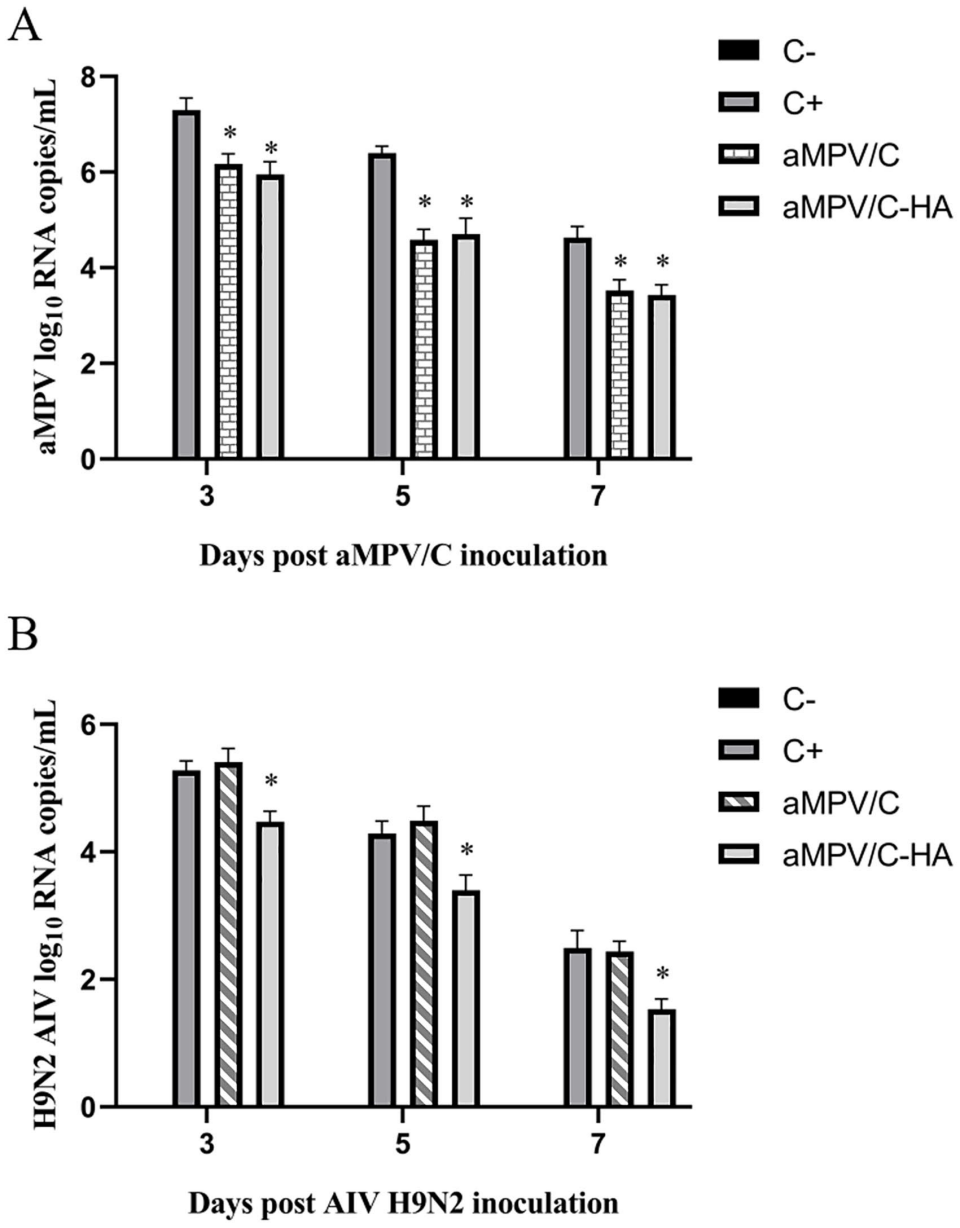
H9N2 AIV and aMPV shedding in trachea of the chickens was detected by qRT-PCR. After 28 dpi of immunization, aMPV and H9N2 AIV were challenged, respectively. Tracheal swabs were collected at 3, 5, and 7 days after the challenge, and the shedding of H9N2 AIV **(A)** and aMPV **(B)** were detected by qRT-PCR. C+: In the positive control group, the aMPV or H9N2 AIV was challenged after PBS was injected into the nose and eye. C−: In the negative control group, there was no challenged after receiving PBS through nose and eye drops. Data presented are the means ± standard deviations (S.D.) from 10 birds in each group. The differences between groups were analyzed using ANOVA (*p < 0.05).

Analysis of the qRT-PCR results further confirmed the protective effect of the vaccine. In the aMPV challenge experiment, the virus content detected in the tracheal swab of the immune group was significantly lower compared with that of the control group (*p* < 0.01) (Figure 6A). In the H9N2 AIV challenge experiment, the tracheal swab virus content of the chickens vaccinated with raMPV/C-HA was also significantly lower relative to that of the chickens vaccinated with aMPV/C and non-immunized groups (*p* < 0.01) (Figure 6B).

## 4 Discussion

Studies have demonstrated that proteins encoded by the paramyxovirus genome possess distinct translation initiation and termination regions, with their structural mRNA precursors synthesized sequentially in a gradient from 3′ to 5′ during viral genome transcription (Huang et al., 2003). The expression levels of individual proteins generally diminishes as the distance from the 3′ end increases. Research has demonstrated that varying insertion sites for foreign genes not only influence their expression but also impact the replication of recombinant viruses. At present, there are few researches on the optimal insertion site of foreign gene in aMPV genome. Falchieri inserted Infectious bronchitis virus (IBV) QX genes into the M-F site of aMPV. A recombinant aMPV-IBV virus expressing IBV QX gene with good immunogenicity was obtained (Falchieri et al., 2013). Notably, researchers have inserted EGFP at five distinct locations within the viral genome and successfully rescued the corresponding recombinant virus. By examining the EGFP expression and virulence characteristics of the recombinant virus, they found that the insertion at the midpoint of the P-M protein was the best site for foreign gene integration (Zhao et al., 2015; Yoshida and Samal, 2017).

In this study, EGFP and H9N2 AIV HA were inserted into the P-M locus of the aMPV genome as exogenous genes, respectively. In addition, we constructed recombinant raMPV/C-EGFP and raMPV/C-HA viruses expressing EGFP and HA. Since the insertion site of the foreign gene is not performed near the F protein, the foreign protein cannot fuse with the transmembrane domain, and therefore, the constructed raMPV/C-HA recombinant virus lack hemagglutination activity (Shirvani et al., 2018).

The efficiency of virus rescue is highly influenced by the ratio of plasmid transfection and the performance of each plasmid involved in the process. The development of mini genome system reduces the difficulty of molecular manipulation of viruses with large genomes, and can also effectively and rapidly conduct pre-experiments, which are mutually experimentally confirmed at the level of recombinant viruses (Lee et al., 2000). Therefore, a mini-genome expressing EGFP was constructed to optimize the conditions for virus rescue.

To optimize the expression of foreign genes while minimizing the impact on recombinant virus replication, the EGFP and HA genes were strategically inserted into the P-M region of the aMPV genome (Qiao et al., 2021; Xu et al., 2019). Analysis of the growth curve of recombinant virus also confirmed that the proliferation of raMPV/C-HA and raMPV/C-EGFP was slightly decreased compared with that of the parent virus, but not significantly.

The aMPV is a highly stable virus which also avoids foreign gene mutations in the passage of recombinant viruses (Cubillos et al., 1991). After 20 generations of the recombinant virus, there was no mutation of the foreign gene.

The different generations of the recombinant viruses were examined using WB and IFA methods, which revealed that the expression of foreign genes did not decrease with the increase of generations. This suggested that aMPV/C is a good recombinant virus vector, with potential to stably express foreign genes during passage. This characteristic is ascribed to the special structure of single-stranded negative RNA viruses, because single-stranded positive RNA viruses are less genetically stable (Cavanagh, 2007).

Results from the experiments confirmed that the raMPV/C-HA elicited significant production of neutralizing antibodies against aMPV and H9N2 AIV in chickens 21–28 days after immunization. The titers of anti-AMPV neutralizing antibodies generated by the raMPV/C-HA immunized chickens were comparable to those produced by the aMPV/C parent virus, indicating that the recombinant virus maintained its autogenicity while expressing foreign genes. Other studies on recombinant aMPV viruses have demonstrated that recombinant viruses do not efficiently induce the production of significant antibodies titers (Falchieri et al., 2013).

Therefore, this is the first study to demonstrate that the aMPV recombinant viruses can stimulate the production of high titer neutralizing antibodies.

Following the challenge of chickens with aMPV and H9N2 AIV, it was observed that the raMPV/C-HA immune flocks were well protected. Only one or two chickens exhibited mild symptoms, which resolved in 3–5 days. The incidence of non-immunized chickens was estimated to be nearly 100%, and all showed the clinical signs of H9N2 AIV or aMPV infection. Given that H9N2 AIV and aMPV are respiratory diseases and their viral breeding area is the upper respiratory tract, tracheal swabs were collected for viral tests. The viral load in chickens immunized with raMPV/C-HA was significantly lower compared to the control group, further confirming the strong protective efficacy of raMPV/C-HA against both aMPV and H9N2 AIV infections. H9N2 AIV is not only transmitted from bird to bird, but can also infect humans, similar to aMPV/C. Notably, aMPV/C has a high homology with hMPV (Luo et al., 2009), and the helper plasmids can be used interchangeably to rescue aMPV/C and hMPV in reverse genetic systems, demonstrating the commonality of aMPV/C and hMPV, which also increases the possibility of aMPV/C infecting humans (Govindarajan et al., 2006). Collectively, these studies underscore the importance of developing interventions to prevent aMPV/C and H9N2 AIV infections.

In conclusion, our study constructed recombinant virus raMPV/C-HA expressing H9N2 AIV HA and analyzed the growth characteristics and immune potency of the recombinant virus. Through the challenge experiments with aMPV and H9N2 AIV in chicken, it was observed that raMPV/C-HA stimulated the secretion of neutralizing antibodies against aMPV and H9N2 AIV, and reduced the proliferation of viruses in the body, conferring good protection. raMPV/C-HA holds great promise to be a candidate vaccine against aMPV and H9N2 AIV.

## Data Availability

The original contributions presented in the study are included in the article/Supplementary material, further inquiries can be directed to the corresponding authors.
