# Supplementary material for: Recombinant avian metapneumovirus subtype C expressing HA protein of H9N2 avian influenza virus are stable and induce protection
Source: Front Microbiol. 2024 Dec 18;15:1513474. doi: 10.3389/fmicb.2024.1513474 (PMC11688360; doi:10.3389/fmicb.2024.1513474)
Supplement: Supplementary file 1 [file Table_1.DOCX]

H9N2 AIV HA

GenBank: MK552998.1

atggagacag tatcactaat aactatacta ctagcagcaa cagtaggcaa tgcagataaa

61 atctgcatcg gctatcaatc aacaaactcc acagaaactg tggacacgct aactgaaaac

121 aatgtccctg tgacacatgc caaagaactg ctccacacag agcataatgg gatgctgtgt

181 gcaacaagct tgggacaacc tcttatttta gacacctgca ccattgaagg gctaatctat

241 ggcaatcctt cctgtgatcc actgccggaa gaaagagaat ggtcctatat cgtcgagaga

301 ccatcagctg ttaacggatt gtgttatccc gggaatgtag aaaatctaga agagctaagg

361 tcacttttta gttctgctag atcttatcaa agaatccaga ttttcccaga cacaatttgg

421 aatgtgtctt acgatgggac aagcaacaca tgctcaggtt cattctacag aaacatgaga

481 tggttgactc gaaaggacgg caattaccct attcaagacg cccaatacac aaataatcaa

541 gggaagaaca ttcttttcat gtggggcata aataacccac ccaccgatga tacgcaaaga

601 aatctgtaca ctagaaccga cacaacaacg agtgtggcaa cggaagaaat aaataggatc

661 ttcaaaccat tgatagggcc aaggcctctt gtcaacggtt tgatgggaag aattaattat

721 tattggtcgg ttttgaaacc gggtcaaaca ctgcgaataa aatctgatgg gaatctagta

781 gctccatggt atggatacat tctttcagga gagagccacg gaagaattct gaggactgat

841 ctaaaaaggg gtagctgcac agtgcaatgt cagacagaga aaggtggctt aaacacaaca

901 ttgccattcc aaaatgtaag taagtatgca tttggaaact gctcaaaata cattggaata

961 aagagcctca aacttgcagt tggtctgagg aatgtgcctt ctagatctag tagaggacta

1021 tttggggcca tagcagggtt tatagaggga ggttggtcag gactagttgc tggttggtat

1081 gggttccagc attcaaatga ccaaggggtt ggtatggcag cagatagaga atcaactcaa

1141 aaggcagttg ataaaataac atccaaagtg aataatatag tcgacaaaat gaacaagcaa

1201 tatgaaatca ttgaccatga attcagtgag gtagaaacta gactcaacat gatcaacaat

1261 aagattgatg atcaaatcca ggatatatgg gcatataatg cagaattgtt agttctgctt

1321 gaaaaccaga aaacactcga tgaacatgac gcaaatgtaa acaatctata taataaagta

1381 aagagagctt tgggttccaa tgcggtggaa gacgggaaag gatgtttcga gctataccac

1441 aaatgtgatg accaatgcat ggagacaatt cggaacggga cttataacag aaggaaatat

1501 caagaggagt caaaattaga aagacagaaa atagaggggg tcaagctgga atctgaagga

1561 acttacaaaa ttctcaccat ttattcgact gtcgcctcat ctcttgtgat tgcaatgggg

1621 tttgctgcct ttttgttctg ggccatgtcc aatgggtctt gcagatgcaa tatttgtata

1681 taa
