# Supplementary figures and images for: Recombinant avian metapneumovirus subtype C expressing HA protein of H9N2 avian influenza virus are stable and induce protection
Source: Front Microbiol. 2024 Dec 18;15:1513474. doi: 10.3389/fmicb.2024.1513474 (PMC11688360; doi:10.3389/fmicb.2024.1513474)

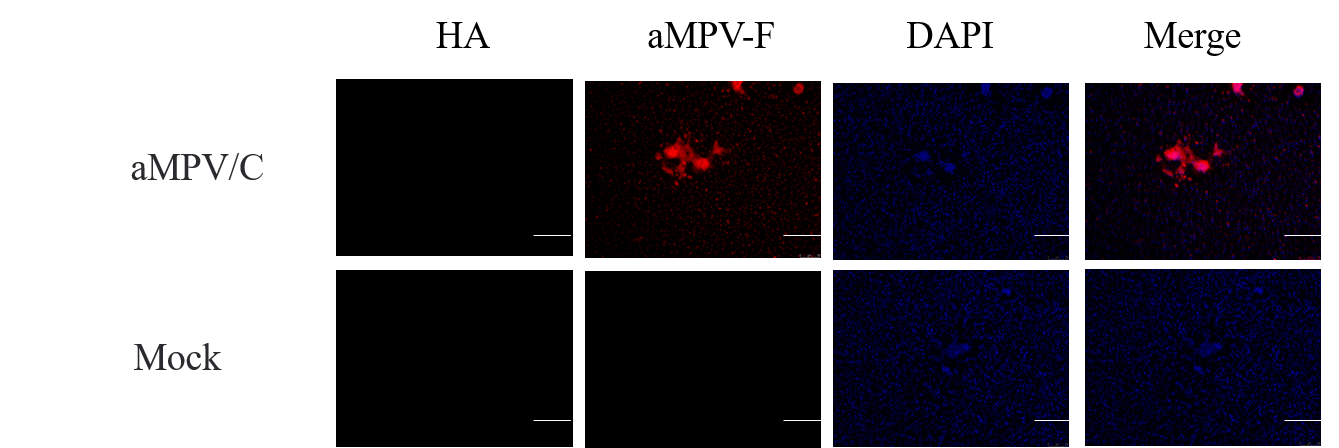

Supplement: Supplementary file 2 [file Data_Sheet_1.ZIP › IFA-MOCK.png]

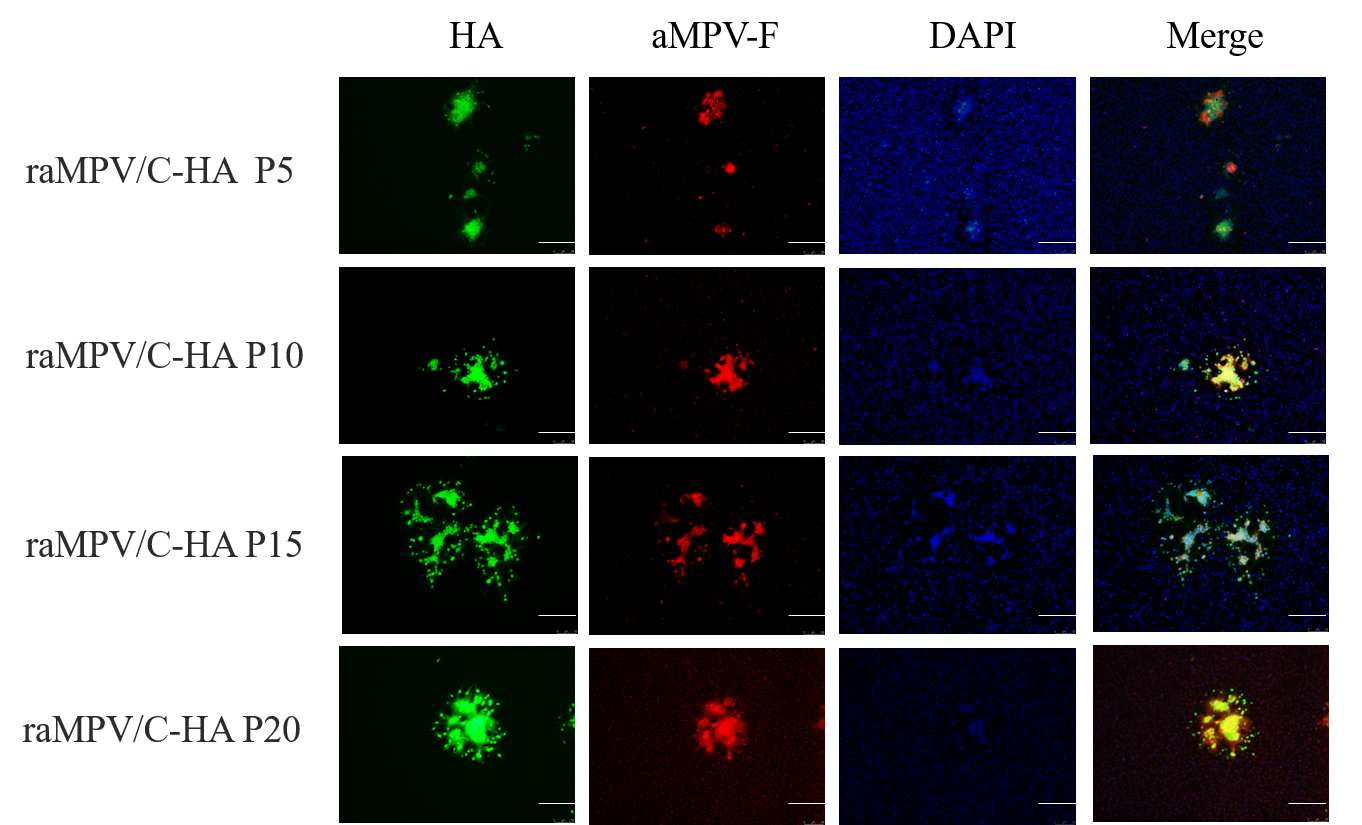

Supplement: Supplementary file 2 [file Data_Sheet_1.ZIP › IFA-EGFP-HA.png]

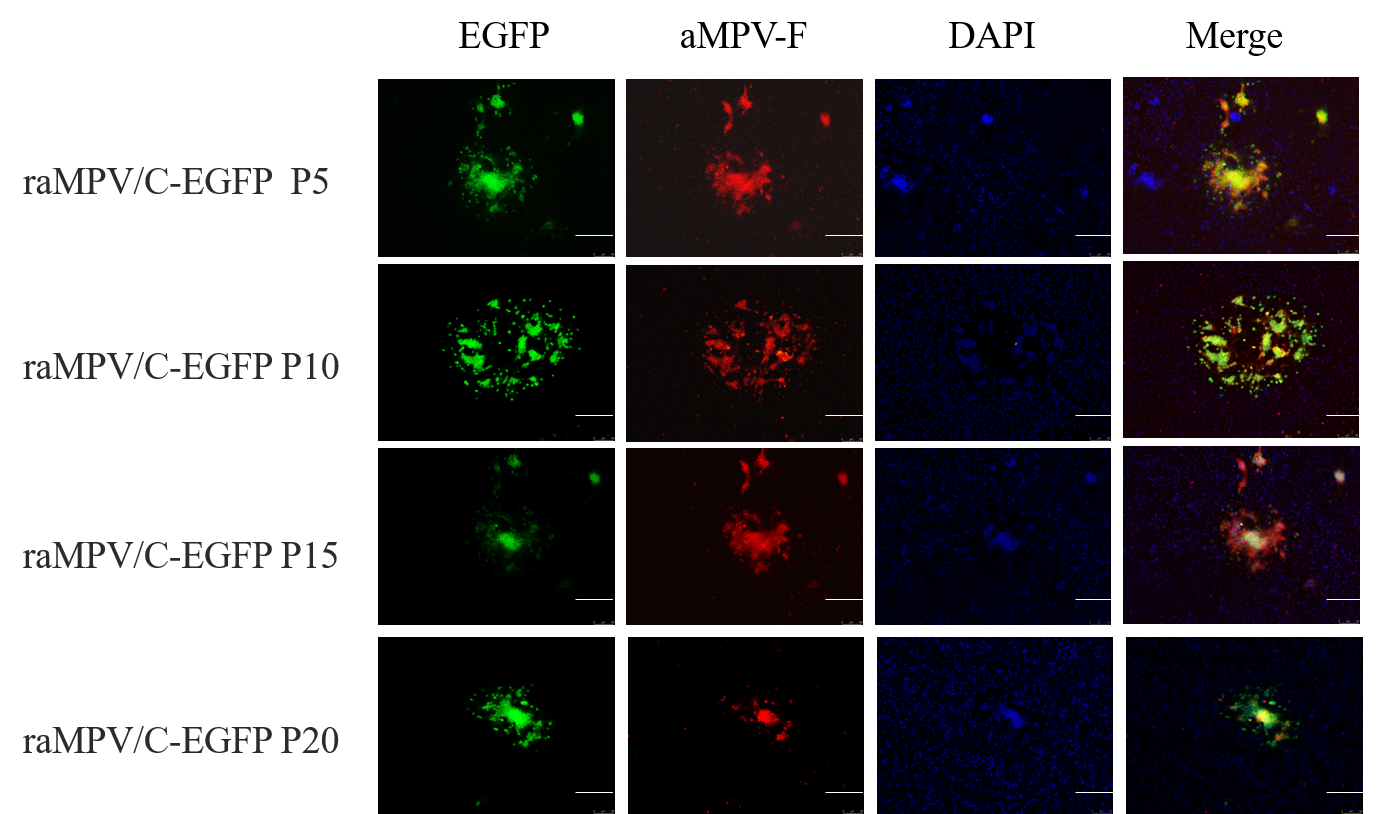

Supplement: Supplementary file 2 [file Data_Sheet_1.ZIP › IFA-AMPV-EGFP.png]

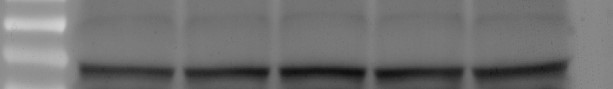

Supplement: Supplementary file 2 [file Data_Sheet_1.ZIP › ACTIN.jpg]

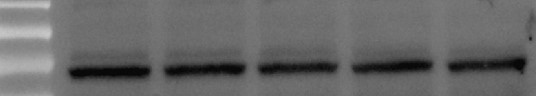

Supplement: Supplementary file 2 [file Data_Sheet_1.ZIP › aMPV-F.jpg]

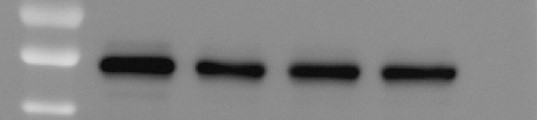

Supplement: Supplementary file 2 [file Data_Sheet_1.ZIP › HA.jpg]
